# Association Between Consumption of Energy and Sports Drinks with Oral Health: A Systematic Review

**DOI:** 10.3390/dj14060359

**Published:** 2026-06-10

**Authors:** Bella Weijia Luo, Nicky Linlin Liang, Ivy Guofang Sun, Chun Hung Chu, Duangporn Duangthip

**Affiliations:** 1 Faculty of Dentistry, The University of Hong Kong, Hong Kong SAR, China; bellaluo@hku.hk (B.W.L.); ivysun24@hku.hk (I.G.S.); chchu@hku.hk (C.H.C.); 2 Department of Health and Exercise Science, University of Oklahoma, Norman, OK 73019, USA; linlin.liang-1@ou.edu; 3 Division of Dental Public Health, College of Dentistry, The Ohio State University, 305 W. 12th Ave, 3166 Postle Hall, Columbus, OH 43210, USA

**Keywords:** dental caries, dental erosion, oral health, sports drinks, energy drinks

## Abstract

**Objectives**: This study aimed to systematically review the association between the consumption of energy drinks and sports drinks with oral health. **Methods**: A systematic search was conducted in three databases (PubMed, Web of Science and Embase) without any restrictions on publication year. Original studies (clinical trials, cohort, case-control studies and cross-sectional studies) reporting the association between energy drink or sports drink consumption and oral health or its impact on oral health were included. **Results**: The initial search found 1196 studies, and 10 studies with a total of 5805 participants (6–89 years old) met the inclusion criteria for analysis. Six studies investigated the association between energy drinks or sports drinks and dental erosion, whereas two studies reported dental caries outcomes and two studies reported saliva outcomes. Most (70%, 7/10) of the included studies were at high or serious risk of bias. Increased frequency or higher amounts of energy drink consumption were associated with a greater risk of dental erosion, whereas the relationship between sports drinks and dental erosion was inconsistent. Evidence regarding the association between dental caries experience and energy drink consumption was controversial, and no significant differences in caries experience were observed for sports drink consumption. **Conclusions**: There was limited and low-quality evidence suggesting that consumption of energy drinks may be potentially associated with dental erosion experience, whereas findings related to dental caries were inconclusive. The association between sports drinks and dental erosion was inconsistent and supported by limited evidence. More well-designed studies on energy or sports drinks and oral health are needed to clarify these relationships.

## 1. Introduction

Nowadays, many individuals have become increasingly reliant on a variety of beverages to help maintain their energy levels and enhance their physical performance. Energy drinks and sports drinks have gained significant popularity in recent years. The appeal of these beverages extends to a broad range of consumers, from athletes to students to professionals, all seeking an extra boost to help them keep up with their demanding schedules and performance expectations. The Food and Drug Administration’s (FDA) classification system does not provide information related to the difference between energy drinks and sports drinks. These terms are instead utilized by beverage companies as marketing strategies to drive sales and appeal to consumers. Typically, both energy drinks and sports drinks contain ingredients such as water, electrolytes, B-vitamins, sugar, or artificial sweeteners with the specific intention of helping consumers engage in physical activity. They work to replenish electrolytes, maintain hydration, and provide a source of energy, all of which are vital during periods of sustained physical exertion. However, energy drinks typically contain stimulants—most notably caffeine—that are not commonly found in sports drinks. Caffeine is a powerful substance that can have a significant impact on the body, providing a temporary boost in energy and mental alertness [1]. The inclusion of caffeine in energy drinks sets these beverages apart from sports drinks in a significant way. While sports drinks are primarily aimed at hydrating the body and replenishing lost electrolytes during physical activity, energy drinks lean more towards improving mental alertness and reducing feelings of tiredness.

In the United States, energy drinks are regulated as food products rather than dietary supplements, which means they are generally available to consumers of all ages, including children and adolescents [2]. Academic research has increasingly focused on both the consumption patterns and the potential health risks associated with energy drinks. Concerns have been raised about excessive caffeine intake and other stimulant-related effects. Their findings underscore that the long-term health consequences—such as cardiovascular stress and sleep disturbances—warrant careful consideration and further study [3].

Besides potential adverse effects on general health, the high sugar content in both energy drinks and sports drinks has raised some health concerns. Energy drinks and sports drinks are often consumed in large quantities and sipped over extended periods, which can lead to prolonged exposure of the teeth to these sugars. The high sugar content and high frequency of consumption provide a readily available source of fermentable sugars for these bacteria, enhancing their ability to produce acids and thus increasing the risk of dental caries [4,5].

Moreover, the acidic components found in energy drinks and sports drinks may lead to another oral health issue known as dental erosion. Dental erosion is a condition characterized by the progressive loss of tooth substance due to acid dissolution. Unlike dental caries, which involves the action of bacteria, dental erosion is a direct result of acid contact with the teeth. When the pH level in the mouth drops below a certain point, the acids in the mouth can start to dissolve the minerals in the tooth enamel, leading to erosion [6].

Despite the growing concern about the potential impact of energy drinks and sports drinks on oral health, a PubMed search conducted on 1 May 2025 revealed that only a limited number of systematic reviews [7,8] examined the relationship between these beverages and dental erosion. However, no systematic review has explored the broader relationship between the consumption of these beverages and oral health outcomes, including dental caries, dental erosion, and pH levels of saliva/plaque. This gap in the literature is particularly concerning given the widespread and increasing consumption of these beverages worldwide and the potential implications for public health. This review aimed to address this gap by providing a comprehensive overview of the existing research on this topic and highlighting the potential oral health risks associated with the consumption of energy drinks and sports drinks.

## 2. Materials and Methods

This systematic review was conducted following the guidelines of the Cochrane Handbook for Systematic Reviews of Interventions Version 6.4 [9] and presented based on the recommendations of the Preferred Reporting Items for Systematic Reviews and Meta-Analysis (PRISMA) statement [10] (Supplementary Materials). The review has been registered in the International Prospective Register of Systematic Reviews (PROSPERO) and the registration number is CRD42024571686.

### 2.1. Search Strategy

A systematic search was conducted in three databases (PubMed, Web of Science, and Embase) to identify all relevant studies published in English. The search was completed by 31 December 2024. The key search terms “sports drinks, energy drinks, oral health, dental caries, dental erosion” were used in this review (Appendix A).

### 2.2. Search Selection

The title and abstract of the identified publications were independently screened by two reviewers (WL and LL). The reference lists of the related studies were also screened to identify all possible eligible studies. Full texts of all potentially relevant publications were obtained and independently reviewed after the screening.

The inclusion criteria for selecting randomized controlled trials (RCTs), controlled clinical trials (CCTs) or observational studies (cohort studies, case-control studies and cross-sectional studies) in this systematic review were as follows, according to PICO guides for RCTs/CCTs or PECO guides for observational studies:

PICO:

Population (P): participants aged 6 or above who have permanent teeth.

Intervention (I): consuming energy drinks or sports drinks.

Comparator (C): negative (no intervention), consuming placebo or positive (alternative intervention) control.

Outcome (O): new dental caries development, dental erosion development assessed via clinical examination, or pH changes in plaque/saliva samples from baseline to endpoint time.

PECO:

Population (P): participants aged 6 or above who have permanent teeth.

Exposure (E): consumption of energy drinks or sports drinks.

Comparator (C): not consuming energy drinks or sports drinks.

Outcome (O): dental caries prevalence, dental erosion prevalence assessed via clinical examination, pH values in plaque/saliva samples.

The exclusion criteria were: (1) children with primary teeth only and participants who do not have permanent teeth; (2) no clinical data reported; (3) no report of the relationships between energy drinks or sports drinks and outcomes.

**Figure 1:**
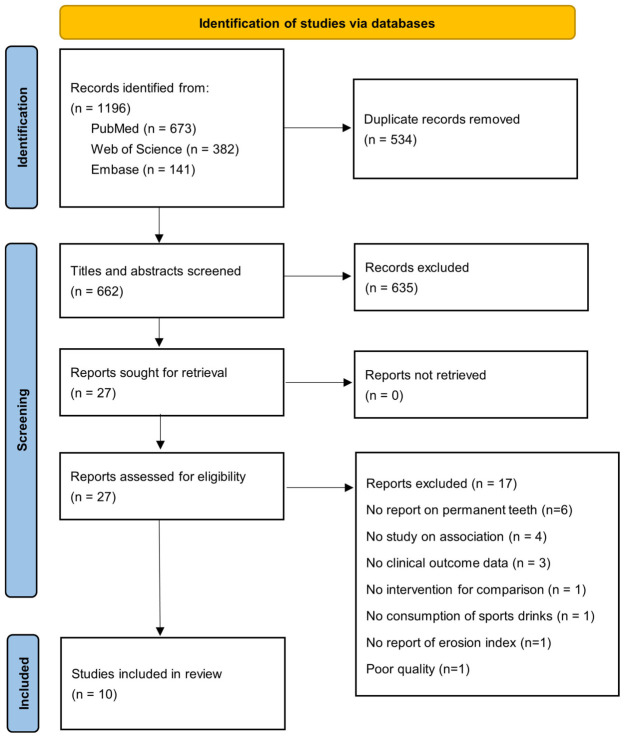
PRISMA 2020 flow diagram.

The final decision about inclusion was made based on the full text of the potentially relevant studies. The process was independently conducted by two reviewers (WL and LL). If no consensus was reached, a third reviewer (DD) was consulted for the final decision. The reasons for exclusion were recorded. Details of the study selection process and the elimination of studies are illustrated in Figure 1.

### 2.3. Risk of Bias Assessment of Included Studies

For RCTs, the Cochrane Collaboration’s risk-of-bias assessment tool (Risk of Bias 2) [11] was used to assess the risk of bias, including bias arising from the randomization process, deviations from intended interventions, missing outcome data, measurement of the outcome, and selection of the reported result. The overall risk of bias was categorized as high, some concerns or low.

For CCTs, the Risk of Bias In Non-randomized Studies of Interventions (ROBINS-I) [12] was used to assess bias, encompassing confounding, selection of participants, classification of interventions, deviations from intended interventions, missing data, measurement of the outcome, and selection of the reported result. The overall risk of bias was classified as no information, critical, serious, moderate, or low.

For observational studies, including cohort studies, case-control studies and cross-sectional studies, Risk of Bias In Non-randomized Studies of Exposure (ROBINS-E) [13] was used to assess the risk of bias (risk of bias due to confounding or arising from measurement of the exposure, selection of participants in the study or in the analysis, post-exposure interventions, missing data, measurement of the outcome, and selection of the reported result). The overall risk of bias was classified as very high, high, some concerns or low.

Two reviewers (WL and LL) independently evaluated the risk of bias in each included study. Consensus was reached by discussion between the two reviewers or by consulting a third reviewer (DD). A summary assessment of the risk of bias in each domain and overall bias was provided for each included study.

### 2.4. Data Synthesis and Outcomes Evaluation

The characteristics of the included studies are summarized in Table 1, Table 2 and Table 3. Extracted data were compared. Meta-analysis may or may not be conducted based on the variance of included studies. For RCTs and CCTs, clinical data about dental caries incidence, dental erosion incidence or pH changes in plaque/saliva samples from baseline to endpoint were extracted. For observational studies, clinical data about dental caries prevalence, dental erosion prevalence or pH values in plaque/saliva samples were extracted.

## 3. Results

A total of 1196 records written in English were identified across three electronic databases (PubMed, Web of Science and Embase). Study screening and data extraction were independently performed by two reviewers (WL and LL). The study flow diagram is shown in Figure 1. Among the identified studies, 534 duplicates were removed. After screening the titles and abstracts, 27 studies were selected for full-text assessment to determine eligibility. Out of these 27 studies, 17 studies were excluded for various reasons, including lack of clinical data, absence of reporting permanent teeth, lack of looking into relationships or other reasons listed in Figure 1. Finally, 10 studies were deemed eligible and included in the review. They comprised eight cross-sectional studies, one RCT, and one CCT study.

### 3.1. Characteristics of Included Studies

All 10 included studies were synthesized and summarized in Table 1, Table 2 and Table 3.

Across the eight cross-sectional studies, one RCT and one CCT study, a total of 5805 participants aged 6 to 89 years having permanent teeth were included. One-third (30%, 3/10) of the studies were conducted in children under 15 years old, whereas more than half (7/10) involved adolescents aged 15 years or older. Half of the studies were conducted in Europe (50%, 5/10), while the remaining were conducted in Asia (40%, 4/10) and America (10%, 1/10).

Sports drinks and energy drinks were identified as two different exposures in three studies [14,17,20], while the remaining seven studies identified them as the same exposure but chose one term (either sports drinks or energy drinks) for reporting. For measuring exposure, different categories were recorded in these included studies, such as “High quantity” versus “Low quantity” [14,15,20], “High frequency” versus “Low frequency” [20] or “Yes” versus “No” [16,17,18,19,21].

For the outcome measurement, caries prevalence (DMFT > 0) was used to evaluate dental caries experience, whereas the dental erosion experience was recorded in different measurements with different indexes, including erosion prevalence, erosion risk prevalence or erosion positive prevalence using the O’Sullivan index, Visual Erosion Dental Examination scoring system, Smith and Knight tooth wear index, Fares index, or Basic Erosive Wear Examination index.

Due to a wide variation in exposure and outcome measurements, as well as the use of different indexes, the outcome data on dental caries experience or dental erosion experience could not be grouped and combined into a meta-analysis. No meta-analysis could be conducted in this study.

Among the eight cross-sectional studies, confounding factors such as dietary habits, oral health practices, and socioeconomic background were considered to varying degrees. Two studies controlled for these factors in the study design (random sampling method to recruit the participants) and adjusted for them in data analyses [19,20], while one study adjusted for them only in data analyses [21]. The remaining studies [14,15,16,17,18] did not control for the confounding factors either in study design or data analyses.

### 3.2. Data Synthesis

In this review, a summary of data from all included studies was organized into three tables based on the outcomes of dental erosion, dental caries and saliva-related measures.

Table 1 summarizes the studies with respect to the outcome of dental erosion (cross-sectional studies);

Table 2 summarizes the studies with respect to the outcome of dental caries (cross-sectional studies);

Table 3 summarizes the studies with respect to the outcome of saliva pH or volume (RCTs and CCTs).

Due to the diverse exposure measurements and outcome measures, data from these included studies could not be appropriately combined for meta-analyses.

Table 1: outcome of dental erosion (cross-sectional studies)

Sports drinks

Four studies [14,15,16,17] reported the association between the consumption of sports drinks and dental erosion experience. The results, measured as erosion prevalence using different indexes, were inconsistent across the four studies. One study [15] reported a significant association, while two studies [14,16] reported no significant difference. Another study [17] presented mixed findings, reporting significant associations in certain age groups—15–29, 30–39, 40–49 and 60–69 years—while other age groups (50–59 and 70–89 years) showed no significant association.

Energy drinks

Four studies [14,17,18,19] reported the association between energy drinks and erosion experience using varying outcome measures, such as erosion prevalence, erosion risk prevalence or erosion positive prevalence. Despite differences in assessment methods, the results were largely consistent. All four studies found that participants who consumed energy drinks [17,18,19] or consumed them in high quantities [14] had a significantly higher risk of developing dental erosion compared to those who did not consume or consumed them in low quantities.

Table 2: outcome of dental caries (cross-sectional studies)

Sports drinks

Only one study [20] reported the association between caries prevalence and exposure to sports drinks. No significant difference was found between participants who consumed sports drinks at high frequency or in large quantities and those with a lower level of consumption.

Energy drinks

Two studies [20,21] reported the association between caries prevalence and the consumption of energy drinks. However, they categorized exposure or energy drinks consumption differently. One [20] compared high-frequency and high-quantity consumption with low-frequency and low-quantity consumption, while the other [21] categorized the exposure into “Yes” or “No” groups.

The results of these two studies were conflicting. One study [20] reported a significant association between the high-frequency or high-quantity consumption of energy drinks and caries prevalence, while the other study [21] reported no significant difference between exposure and non-exposure groups.

Table 3: outcome of saliva pH or volume (RCT and CCT).

One RCT study [22] and one CCT study [23] investigated the association between the consumption of sport drinks and salivary parameters, including volume or pH. The findings on saliva pH changes were inconsistent between these two studies, while no significant difference was found in saliva volume following sports drinks consumption [22].

### 3.3. Risk of Bias Assessment

**Figure 2:**
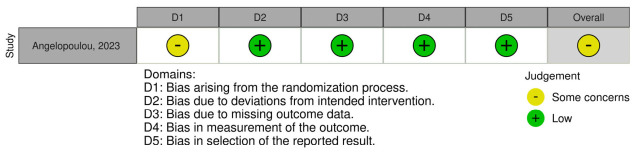
Risk of bias assessment of included RCTs (RoB 2 assessment tool) [22].

**Figure 3:**
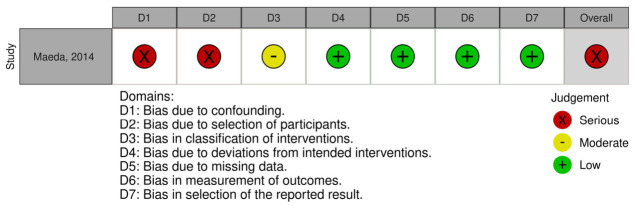
Risk of bias assessment of included CCTs (ROBINS-I assessment tool) [23].

**Figure 4:**
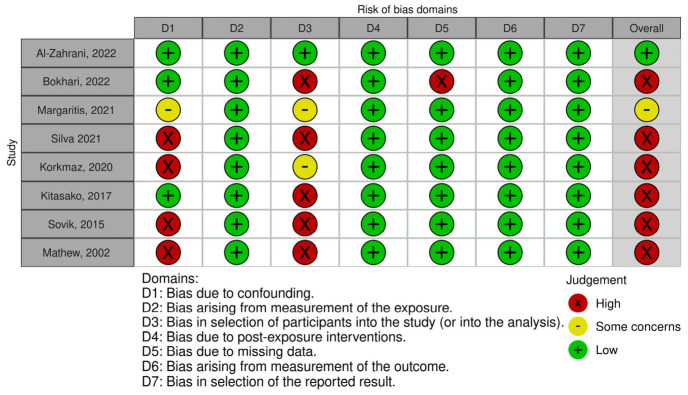
Risk of bias assessment of included cross-sectional studies (ROBINS-E assessment tool) [14,15,16,17,18,19,20,21].

The risk of bias assessment for the included studies is presented in Figure 2, Figure 3 and Figure 4. One RCT [22] and one cross-sectional study [19] were assessed as having some concerns. One CCT [23] and six cross-sectional studies [14,15,16,17,18,21] were judged to be at high or serious risk of bias. Only one cross-sectional study [20] was considered to be at low risk of bias.

## 4. Discussion

Recently, energy drinks and sports drinks have attracted growing attention in academic research due to their widespread consumption, potential health effects, and implications for physical and cognitive performance. Results of the current study indicate that relatively few studies have examined the relationship between energy drinks or sports drinks and oral health conditions. Among the included studies, the majority focused on the relationship between their consumption and dental erosion. Notably, studies investigating the association between erosion prevalence and sports drinks reported inconsistent findings, which is consistent with a previous systematic review [7], whereas another review reported that no association was found between sports drinks and dental erosion [8].

In contrast, studies assessing erosion prevalence in relation to energy drinks consistently reported a potential association, even when employing various measurements to record the dental erosion condition. This may be due to the compositional differences between energy drinks and sports drinks. While the primary additional ingredient in energy drinks is caffeine, current evidence does not support a direct role with respect to caffeine in the development of dental erosion. The reasons underlying this discrepancy remain unclear, underscoring the need for future research to explore other potential contributing factors. On the other hand, the evidence on the relationships between energy drinks or sports drinks and dental caries or saliva parameters (pH and volume) remained inconclusive, due to the limited number of studies and inconsistent findings.

It is also essential to critically discuss the definition and distinction between energy drinks and sports drinks in academic research. During our systematic search, these terms appeared interchangeable across major databases such as PubMed, Web of Science, and Embase, where both terms are indexed under the same MeSH term “energy drinks”. However, upon reviewing the included studies, we observed variation in how these beverages were classified. Some authors clearly differentiated between energy drinks and sports drinks as separate exposures in their study design and questionnaires [14,17,20], while others treated them as a single category. It remains uncertain whether participants in these studies fully understood or distinguished between the two beverage types when self-reporting consumption. This potential confusion highlights a methodological challenge in dietary exposure research. Therefore, we recommend that future studies clearly define and distinguish between energy drinks and sports drinks and consider validating participant understanding to improve the accuracy and reliability of reported data.

Dental erosion has emerged as a growing area of research, particularly due to the increasing global consumption of energy and sports drinks [24]. Energy drinks or sports drinks often contain high levels of acids, such as citric acid and phosphoric acid, which can erode tooth enamel over time. The process can be relatively short in minutes (early sign) or very long in years (visible change) [25,26], which is determined by various factors, such as acid frequency, duration and strength, as well as oral hygiene, saliva, or individual susceptibility. One of the key challenges in studying dental erosion may be the lack of a clearly defined threshold for acid exposure. No specific duration or frequency has been conclusively linked to the onset of dental erosion. This makes it difficult to design and interpret erosion studies with precision. In this review, all included studies reporting dental erosion were cross-sectional studies, and the quality of included studies was low. Further high-quality prospective cohort studies with extended follow-up periods should be conducted to systematically investigate the multifactorial etiology of dental erosion, with particular emphasis on quantifying dose-response relationships and identifying potential modifying factors.

Unlike dental erosion, dental caries is a multifactorial dynamic disease involving the interaction of cariogenic bacteria with dietary sugars, time, and tooth surface. While sugar intake is a recognized caries risk factor, it is not a solo pathogenic factor. Therefore, the disparity in sugar content between energy drinks and sports drinks is unlikely to yield significant outcomes in the development of dental caries. This is because caries formation depends on a complex interplay of factors beyond just sugar concentration. Furthermore, all the included studies reporting on dental caries were cross-sectional in nature, and the overall quality of these studies was low. To better understand the potential influence of energy drinks and sports drinks on caries development, well-designed prospective cohort studies are needed.

This review identified substantial variations in the measurement tools used to assess dental erosion or erosion risk across the included studies. No single instrument or index was applied consistently. The lack of standardization posed significant challenges for data extraction, synthesis, analysis, and comparison. In addition, wide variability existed in exposure definitions and outcome measurements. Thus, the data could not be meaningfully grouped and combined in a meta-analysis. Conducting a pooled analysis under such conditions would have produced potentially misleading summary estimates. As a result, drawing comparable conclusions across studies was inherently limited. To advance the quality and comparability of future research, we advocate extensive discussion on the standardized measurement tools used for assessing dental erosion outcomes. Establishing a widely accepted and validated tool would enhance the reliability of findings and support the development of higher-quality scientific evidence in this field.

In addition, it should be noted that half of the included cross-sectional studies did not adjust for important confounding factors—such as overall dietary sugar intake, oral hygiene behaviors, socioeconomic status, frequency of acidic beverage exposure, salivary flow, fluoride exposure, and eating disorders or reflux—that may influence the outcomes. As a result, the findings may be subject to some degree of bias.

As for the limitations, most of the included studies were cross-sectional studies. The questionnaire designs may not be consistent across all the studies. Heterogeneity in methodologies across studies further complicates synthesis and introduces variability in quality, while residual confounding factors may distort observed associations if inadequately adjusted. No meta-analysis was performed due to the heterogeneity in study designs, exposure definitions, and outcome measurements, which limited the strength of this review. Another concern is publication bias, where positive or statistically significant findings are overrepresented, potentially skewing pooled estimates and reducing the reliability of conclusions drawn. Only English-language studies were included, which introduces another potential language bias.

While these limitations prevent definitive conclusions, the findings offer significant value by highlighting critical gaps in the current body of research. This study serves as a foundational step toward a more comprehensive understanding of the relationship between energy and sports drink consumption and oral health outcomes, particularly dental erosion and caries. Further research is needed to fully understand the extent of these associations and to develop effective strategies for minimizing the oral health impact of energy or sports drinks consumption.

## 5. Conclusions

There was limited, low-quality evidence suggesting that consumption of energy drinks may be potentially associated with dental erosion, whereas findings related to dental caries or saliva outcomes were inconclusive. The association between dental erosion and sports drinks consumption was inconsistent and supported by limited evidence. More well-designed studies are needed to clarify these relationships.

## Figures and Tables

**Table 1:** Summary of the included cross-sectional studies for dental erosion outcome (*n* = 6).

First Author, Year (Country)	Number of Participants (Age, Years)	Exposure Measurement	Outcome Measurement (Index)	Erosion/Risk/Positive Prevalence		Main Finding	Confounding Factors
				With Exposure	Without Exposure	OR (95%CI) *p*-Value	
Exposure: sports drinks							
Korkmaz, 2020 ^#^ (Turkey) [14]	473 (7–14)	High quantity ^!^	Erosion prevalence (O’Sullivan)	Not reported	Not reported	2.22 (0.56–8.78) 0.252	Unadjusted
Sovik, 2015 (Norway) [15]	795 (16–18)	High quantity ^$^	Erosion prevalence (Visual Erosion Dental Examination scoring system)	54.3%	35.9%	2.12 (1.07–4.20) 0.031 *	Unadjusted
Mathew, 2002 (USA) [16]	304 (18–28)	Yes	Erosion prevalence (Lussi)	36.2%	40%	0.85 (0.37–1.96) 0.705	Unadjusted
Kitasako, 2017 ^@^ (Japan) [17]	191 (15–29)	Yes	Erosion positive ^ prevalence (Smith and Knight tooth wear index and Fares index)	48.6%	19.7%	3.87 (2.03–7.38) <0.001 *	Adjusted
	182 (30–39)	Yes		42.6%	19.5%	3.06 (1.53–6.12) 0.002 *	
	189 (40–49)	Yes		42.9%	17.1%	3.63 (1.77–7.42) <0.001 *	
	182 (50–59)	Yes		30.8%	19.2%	1.87 (0.90–3.88) 0.095	
	187 (60–69)	Yes		43.1%	19.1%	3.21 (1.59–6.46) 0.001 *	
	177 (70–89)	Yes		33.3%	25.6%	1.45 (0.71–2.98) 0.307	
Exposure: energy drinks							
Korkmaz, 2020 ^#^ (Turkey) [14]	473 (7–14)	High quantity ^!^	Erosion prevalence (O’Sullivan)	Not reported	Not reported	10.06 (1.34–75.27) 0.024 *	Unadjusted
Silva, 2021 (Portugal) [18]	110 (13–62)	Yes	Erosion risk ^&^ prevalence (BEWE)	Not reported	Not reported	3.34 (1.42–7.87) 0.006 *	Unadjusted
Margaritis, 2021 (Finland, Romania, Greece and USA) [19]	207 (15–21)	Yes	Erosion prevalence (BEWE)	84.9%	49.4%	5.77 (2.55–13.05) <0.001 *	Adjusted
Kitasako, 2017 ^@^ (Japan) [17]	191 (15–29)	Yes	Erosion positive ^^^ prevalence (Smith and Knight tooth wear index and Fares index)	46.0%	25.5%	2.48 (1.27–4.87) 0.008 *	Adjusted
	182 (30–39)	Yes		45.2%	22.5%	2.84 (1.27–6.33) 0.011 *	
	189 (40–49)	Yes		36.4%	22.2%	2.01 (0.78–5.15) 0.147	
	182 (50–59)	Yes		41.7%	19.6%	2.93 (1.19–7.21) 0.020 *	
	187 (60–69)	Yes		42.3%	23.0%	2.46 (1.04–5.81) 0.040 *	
	177 (70–89)	Yes		38.1%	26.3%	1.73 (0.67–4.46) 0.260	

* Statistically significant at *p* < 0.05; OR: odds ratio; CI: confidence interval; ^#^ the same publication; ^@^ the same publication; BEWE: Basic Erosive Wear Examination; ^&^ Erosion risk: BEWE score > 2 was considered ‘‘risk’’ of erosion. ^^^ Erosion positive: subjects having at least one tooth with an initial enamel wear (score 2) were grouped into the erosion positive group. ^!^ On the basis of cups, but no exact cups reported. ^$^ Taking more than 250 mL of sports drinks per day.

**Table 2:** Summary of the included cross-sectional studies for dental caries outcome (caries prevalence: DMFT > 0) (*n* = 2).

First Author, Year (Country)	Number of Participants (Age, Years)	Exposure Measurement	Caries Prevalence		Main Finding	Confounding Factors
			With Exposure	Without Exposure	OR (95%CI) *p*-Value	
Exposure: sports drinks						
Al-Zahrani, 2022 ^#^ (Saudi Arabia) [20]	2262 (12–15)	High frequency ^^^	Not reported	Not reported	1.26 (0.74–2.24) >0.05	Adjusted
		High quantity ^@^	Not reported	Not reported	1 (0.56–1.78) >0.05	
Exposure: energy drinks						
Al-Zahrani, 2022 ^#^ (Saudi Arabia) [20]	2262 (12–15)	High frequency ^	Not reported	Not reported	1.88 (1.22–2.89) <0.05 *	Adjusted
		High quantity ^@^	Not reported	Not reported	1.91 (1.24–2.93) <0.05 *	
Bokhari, 2022 (Saudi Arabia) [21]	448 (18–45)	Yes	Not reported	Not reported	0.64 (0.29, 1.42) 0.27	Adjusted

* Statistically significant at *p* < 0.05; OR: odds ratio; CI: confidence interval; ^#^ The same publication. ^^^ Taking sports drinks or energy drinks daily or more frequently; ^@^ Taking sports drinks or energy drinks more than 299 mL per day.

**Table 3:** Summary of the included RCT or CCT for saliva pH or volume outcome (*n* = 2).

First Author, Year (Country)	Number of Participants (Age, Years)	Study Design	Intervention	Outcome Measurement	Main Finding *p*-Value
Angelopoulou, 2023 (Ireland) [22]	68 (6–14)	RCT	Gp1: sports drinks Gp2: water	Mean saliva pH	After drink: Gp1: 6.63; Gp2: 7.06 0.013 *
				Saliva volume (ml)	After drink: Gp1: 2.92; Gp2: 2.64 0.813
Maeda, 2014 (Japan) [23]	30 (NA)	CCT	Gp1: sports drinks Gp2: no drinking	Saliva pH changes	Gp1 < Gp2 >0.05

* Statistically significant at *p* < 0.05; Gp: group. NA: not available—recruited adults without age reported.

## Data Availability

All data included in this systematic review are publicly available from the original studies cited in the manuscript.

## Supplementary Materials

The following supporting information can be downloaded at: https://www.mdpi.com/article/10.3390/dj14060359/s1, Table S1: PRISMA 2020 Checklist.

## Appendix A

PubMed		
#1	(((energy drinks[MeSH Terms]) OR (spor* drink)) OR (sport drink*)) OR (energy drink*)	18,082
#2	(oral health[MeSH Terms]) OR (dental health)	353,650
#3	(((((dental caries[MeSH Terms]) OR (dental cari*)) OR (tooth decay)) OR (dental cavit*)) OR (tooth cavit*)) OR (cari* lesion)	120,750
#4	(((erosion, tooth[MeSH Terms]) OR (tooth wear[MeSH Terms])) OR (tooth wear)) OR (dental erosi*)	11,518
#5	(((oral health[MeSH Terms]) OR (dental health)) OR ((((((dental caries[MeSH Terms]) OR (dental cari*)) OR (tooth decay)) OR (dental cavit*)) OR (tooth cavit*)) OR (cari* lesion))) OR ((((erosion, tooth[MeSH Terms]) OR (tooth wear[MeSH Terms])) OR (tooth wear)) OR (dental erosi*))	444,912
#6	((((energy drinks[MeSH Terms]) OR (spor* drink)) OR (sport drink*)) OR (energy drink*)) AND ((((oral health[MeSH Terms]) OR (dental health)) OR ((((((dental caries[MeSH Terms]) OR (dental cari*)) OR (tooth decay)) OR (dental cavit*)) OR (tooth cavit*)) OR (cari* lesion))) OR ((((erosion, tooth[MeSH Terms]) OR (tooth wear[MeSH Terms])) OR (tooth wear)) OR (dental erosi*)))	673
Web of Science		
#1	((TS = (energy drink*)) OR TS = (spor* drink)) OR TS = (sport drink*)	18,770
#2	(TS = (oral health)) OR TS = (dental health)	127,718
#3	(((((TS = (dental caries)) OR TS = (dental cari*)) OR TS = (tooth decay)) OR TS = (dental cavit*)) OR TS = (tooth cavit*)) OR TS = (cari* lesion)	61,406
#4	((TS = (tooth erosion)) OR TS = (tooth wear)) OR TS = (dental erosi*)	13,237
#5	#2 OR #3 OR #4	180,511
#6	#1 AND #5	382
Embase		
1	energy drink/or energy drink*.mp.	3398
2	sports drink/or sport drink*.mp. or energy drink/	3613
3	sports drink/or spor* drink.mp.	1137
4	1 or 2 or 3	4415
5	oral health.mp.	41,326
6	dental health.mp. or dental health/	19,737
7	5 or 6	54,902
8	dental caries.mp. or dental caries/	72,959
9	dental caries/or dental cari*.mp.	72,974
10	tooth decay.mp. or dental caries/	69,219
11	dental caries/or dental cavit*.mp.	68,902
12	tooth cavit*.mp.	260
13	dental caries/or cari* lesion.mp.	68,904
14	8 or 9 or 10 or 11 or 12 or 13	74,136
15	tooth erosion.mp. or dental erosion/	862
16	tooth wear.mp. or tooth wear/	2390
17	dental erosion/or dental erosi*.mp.	1738
18	15 or 16 or 17	4090
19	7 or 14 or 18	116,998
20	4 and 19	141
